# Defining *in vitro* topical antimicrobial and antibiofilm activity of epoxy-tigliane structures against oral pathogens

**DOI:** 10.1080/20002297.2023.2241326

**Published:** 2023-07-31

**Authors:** Wenya Xue, Manon F. Pritchard, Saira Khan, Lydia C. Powell, Joana Stokniene, Jingxiang Wu, Nicholas Claydon, Paul Reddell, David W. Thomas, Katja E. Hill

**Affiliations:** a Advanced Therapies Group, Cardiff School of Dentistry, Heath Park, Cardiff University, Cardiff, UK; b Microbiology and Infectious Disease Group, Swansea University Medical School, Swansea, UK; c QBiotics Group Limited, Yungaburra, Queensland, Australia

**Keywords:** Dental implants, antimicrobial therapy, peri-implantitis, titanium, biofilm, epoxy-tiglianes

## Abstract

**Background:**

Peri-implantitis has become an inexorable clinical challenge in implantology. Topical immunomodulatory epoxy-tiglianes (EBCs), derived from the Queensland blushwood tree, which induce remodeling and resolve dermal infection via induction of the inflammasome and biofilm disruption, may offer a novel therapeutic approach.

**Design:**

*In vitro* antimicrobial activity of EBC structures (EBC-46, EBC-1013 and EBC-147) against *Streptococcus mutans*, *Aggregatibacter actinomycetemcomitans* and *Porphyromonas gingivalis* in minimum inhibitory concentration, growth curve and permeabilization assays were determined. Antibiofilm activity was assessed using minimum biofilm eradication concentration (MBEC) experiments. Biofilm formation and disruption assays were analyzed using confocal laser scanning microscopy, scanning electron microscopy and direct plate counting.

**Results:**

The observed antimicrobial efficacy of the tested compounds (EBC-1013 > EBC-46 > EBC-147) was directly related to significant membrane permeabilization and growth inhibition (*p* < 0.05) against planktonic *S.* *mutans* and *P.* *gingivalis*. Antibiofilm activity was evident in MBEC assays, with *S.* *mutans* biofilm formation assays revealing significantly lower biomass volume and increased DEAD:LIVE cell ratio observed for EBC-1013 (*p* < 0.05). Furthermore, biofilm disruption assays on titanium discs induced significant biofilm disruption in *S.* *mutans* and *P.* *gingivalis* (*p* < 0.05).

**Conclusions:**

EBC-1013 is a safe, semi-synthetic, compound, demonstrating clear antimicrobial biofilm disruption potential in peri-implantitis.

## Introduction

Peri-implantitis is characterized by inflammation in the peri-implant mucosa and progressive loss of supportive alveolar bone [1]. An average follow-up of 2 years demonstrated peri-implantitis affected 21% of all implants, accounting for 34% of the patients presenting for treatment [2]. Persisting dental biofilms are the primary aetiological factor that initiate the development of the inflammation in peri-implantitis [3], with the heterogeneous mixed infection and virulence factors causing dysbiosis of the resident microflora and disruption of host-microbe homeostasis [4]. Paradoxically, this induces innate immune system/inflammasome induction, promoting growth of a pathogenic (inflammophilic) microbiota, thereby reducing the effectiveness of inflammation in microbial clearance [5]. Within peri-implant disease sites, the role of Gram-negative anaerobes and asaccharolytic anaerobic Gram-positive rods has been highlighted [6,7]. Furthermore, commensal anaerobic microorganisms are known to effectively disrupt the normal wound repair/remodeling processes of resident cell populations such as keratinocytes, fibroblasts, and endothelial cells [8,9].

Treatment for peri-implantitis is based on addressing the microbial aetiology and is strategically organized to target bacterial plaque accumulation (using oral health advice to improve self-care) as well as treatment (professional intervention) of the inflammatory lesion [10]. However, the mechanical debridement of the affected implants is considered ineffective in reducing signs or symptoms of the disease [10,11]. In consequence, systemic, topical antibiotic and antibacterial therapies have been combined with mechanical debridement to decontaminate the implant surface, reduce the bacterial load and decrease local inflammation/tissue destruction. A recent systematic review demonstrated that of all physical and chemical adjunctive therapies (including antibiotic/antimicrobial/chemical treatment, laser and mechanical therapies), only antibiotic therapy offered any clinical benefits (notably reducing pocket probing depth and radiographic bone loss) in the short term (<6 months), although these effects dissipated in the long term (>12 months) [12].

Epoxy-tiglianes are small diterpene esters derived from the fruit of *Fontainea picrosperma* indigenous to the Australian rainforest which represent a new range of therapeutics, with multi-modal pharmacological properties including immunomodulatory [13], anti-cancer [14] and antimicrobial [15] effects. Their ability to resolve bacterial infection and chronic inflammation via two orthogonal mechanisms has recently been described. Firstly, via direct local immune cell induction mediated by protein kinase C (PKC)-activation in both resident- (keratinocytes and fibroblasts) and migratory cells (neutrophils; PMNLs) [13,16]. Secondly, via direct effects on bacteria and remodeling and re-epithelialization observed in burn injury and diabetic wound models [14,15]. Importantly, the rapid pro-inflammatory stimulus induced by epoxy-tiglianes remains highly localized and self-limiting due to the strongly lipophilic nature of the compound, making it ideal for topical applications. The establishment of a library of natural and semi-synthetic epoxy-tigliane analogues (with differing bioactivities) offers the opportunity to investigate both structure/activity relationships and, test their efficacy in the treatment of other human diseases mediated by biofilm infection and chronic inflammation, such as peri-implantitis.

The aim of this *in vitro* study was to investigate the anti-bacterial and antibiofilm effects of three epoxy-tigliane structures (EBC-1013, EBC-46 and EBC-147) against oral pathogens implicated in peri-implantitis. This preliminary investigation will determine their applicability in the treatment of peri-implantitis and identify a lead candidate for further clinical development.

## Materials and methods

### Microbial culture

Oral pathogens were chosen to compare structure/activity relationships to cell wall architecture and modes of growth. Bacterial strains employed were *Streptococcus mutans* DSM 20523 (ATCC 25175) and *Aggregatibacter actinomycetemcomitans* DSM 8324 (ATCC 33384) demonstrating Gram-positive and Gram-negative facultative anaerobes, respectively, and a Gram-negative obligate anaerobe, *Porphyromonas gingivalis* NCTC 11834 (ATCC 33277). Bacterial colonies of *S. mutans* and *A. actinomycetemcomitans* were maintained on blood agar (BA, Lab M) supplemented with 5% horse blood (ThermoFisher Scientific, Loughborough, UK), brain heart infusion (BHI) broth (ThermoFisher Scientific, Loughborough, UK) or Mueller Hinton (MH) broth (Neogen Europe Ltd., Ayr, UK) and grown microaerophilically (5% CO_2_) at 37°C. *P. gingivalis* was maintained on fastidious anaerobe agar (FAA, ThermoFisher Scientific, Loughborough, UK) supplemented with 5% horse blood or in fastidious anaerobe broth (FAB, ThermoFisher Scientific, Loughborough, UK) and grown anaerobically (10% CO_2_, 10% H_2_, 80% N_2_) at 37°C. Overnight (O/N) *S. mutans* (18–20 h) and *A. actinomycetemcomitans* (48 h) bacterial cultures in BHI and *P. gingivalis* (72 h) cultures in FAB were prepared prior to each experiment.

### Epoxy-tigliane (EBC) compounds

**Figure 1.**
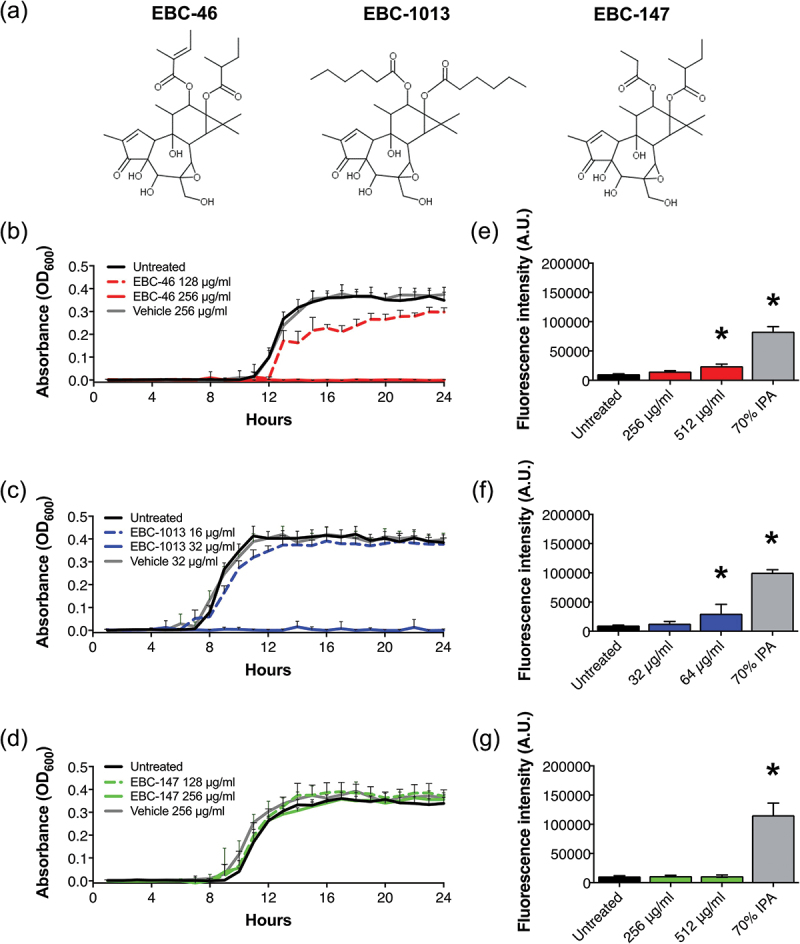
Chemical structure of the epoxy-tigliane test compounds (a) EBC-46, EBC-1013 and EBC-147. *S. mutans* (DSM 20523) growth curves in MH broth (24 h) ± (b) EBC-46, (c) EBC-1013 and (d) EBC-147 with untreated and vehicle equivalent control (v/v). Effect of (e) EBC-46, (f) EBC-1013 and (g) EBC-147 on cell membrane permeabilization of *S. mutans* (DSM 20523), with untreated and 70% isopropanol positive controls. Results are expressed as fluorescence intensity (A.U.; *p < 0.05).

Epoxy-tigliane (EBC) compounds were provided by QBiotics Group Ltd, Yungaburra, Queensland, Australia. The structure of the EBC compounds used in this study varied in relation to the presence of C12 and C13 esters or C12 ester chain length (Figure 1a) and included EBC-46 (tigilanol tiglate), EBC-1013 (semi-synthetic, C12 C13 dihexanoate) and EBC-147 (short C12 ester; included as a control demonstrating comparatively low antibacterial activity in previous studies) [15]. EBCs were reconstituted in ethanol; therefore, ethanol-equivalent (vehicle-only) controls were used for all experiments.

### Minimum inhibitory concentration assays (MICs)

Broth microdilutions were performed according to Jorgensen et al. (2015) [17], with modifications for *P. gingivalis* (O/N cultures adjusted to an OD_600_ of 0.8 in FAB). Plates were incubated for 16–20 h for *S. mutans*, 48 h for *A. actinomycetemcomitans* and 72 h for *P. gingivalis*. Resazurin (0.01% in dH_2_O) was added to each well and plates were incubated for a further 3 h, after which MIC values were determined by visual inspection.

### Minimum biofilm eradication assays (MBECs)

MBEC assays were performed according to Cowley et al. (2015) [18]. The *S. mutans* and *A. actinomycetemcomitans* O/N cultures were diluted to an OD_600_ of 0.08 in MH broth whilst *P. gingivalis* (72 h) cultures were adjusted to an OD_600_ of 0.8 in FAB. Microtitre (96-well) plates were incubated statically for 48 h for *S. mutans*, 72 h for *A. actinomycetemcomitans* or 96 h for *P. gingivalis*. For *P. gingivalis*, spent medium was replaced with fresh FAB after 48 h to assist biofilm formation. Following incubation, supernatants were removed, and the biofilms washed with PBS prior to EBC treatment for 24 h (37°C; statically). After incubation, the supernatants were removed from the biofilms and fresh broth (with no EBC treatment) was added to each well. Plates were then re-incubated for a further 24 h at 37°C prior to further incubation with resazurin (0.01% in dH_2_O) for 3 h. MBEC values were determined by visual assessment.

### Growth kinetics of oral bacteria

O/N cultures were adjusted to OD_600_ of 0.08–0.10 (*S. mutans* [MH broth] and *A. actinomycetemcomitans* [BHI broth]) or 0.8–1.0 (*P. gingivalis* [FAB]). Adjusted cultures were then diluted in 96-well plates in EBCs with untreated and vehicle-only controls (1 in 20) in their respective broths. For *P. gingivalis* plates, an anaerobic environment was generated by the addition of AnaeroGen™ (ThermoFisher Scientific, Loughborough, UK) [19] with silicone grease lid seals (SGM494, ACC® silicones) and the plates wrapped in parafilm. Oxygen elimination was monitored by including an anaerobic indicator test strip (Anaerotest®, Merck, Gillingham, UK) in the plate. Measurements of growth were recorded hourly at OD_600_ after shaking at 200 rpm for 20 s in a FLUOstar® Omega multi-mode microplate reader at 37°C for 24 h (*S. mutans*), 48 h (*A. actinomycetemcomitans*) or 72 h (*P. gingivalis*).

### Membrane permeability assay

Cell permeabilization following EBC treatment (16–512 µg/ml) was determined using the SYTOX™ Green Nucleic Acid Stain (ThermoFisher Scientific, Loughborough, UK) [20]. Positive controls were treated with 70% isopropanol, untreated and vehicle-only controls were also included.

### Confocal laser scanning microscopy (CLSM)

CLSM assays were performed as described by Powell et al., (2018) [21]. *S. mutans* and *A. actinomycetemcomitans* were standardized to OD_600_ of 0.10 (8 × 10^7^ CFU/ml) and *P. gingivalis* to 0.8 (6.4 × 10^8^ CFU/ml), prior to a 1:10 inoculation in BHI broth or FAB ± EBCs and incubated for 24 h for *S. mutans*, 48 h for *A. actinomycetemcomitans* or 96 h for *P. gingivalis* (replacing 50 μl of spent culture medium after 48 h), within Greiner glass bottom optical 96-well plates. After the incubation, the supernatant was removed and the biofilms were stained with 0.7% (v/v) LIVE/DEAD® stain (BacLightTM Bacterial Viability Kit, ThermoFisher Scientific, Loughborough, UK) in phosphate-buffered saline (PBS) for 10 min, prior to imaging. CLSM Z-stack 3D images were taken using a Leica SP5 confocal microscope. Z-stack CLSM images were analyzed using COMSTAT image analysis software [22].

### Biofilm disruption on titanium discs

Overnight cultures were adjusted to OD_600_ of 0.08–0.10 (*S. mutans*) in BHI broth or to 0.8 (*P. gingivalis*) in FAB, then added to the wells of (two identical) 96-well microtiter plates, each well containing a sterile titanium disc (5 mm diameter × 3 mm thick; Goodfellow Cambridge Ltd, Huntington, UK). The plates were incubated for 24 h (*S. mutans*) or 96 h (*P. gingivalis;* replacing spent medium with 100 μl of fresh FAB after 48 h). Following biofilm formation, 100 μl of the supernatant was removed and replaced with BHI broth or FAB ± EBCs (256 μg/ml v/v) with untreated and vehicle-only controls. The plates were then incubated statically for 24 h prior to analysis. Biofilm disruption was analyzed using one plate for scanning electron microscopy (SEM) imaging and the second plate for drop count assays (CFU/ml) of the biofilms coating the titanium surface (*n* = 3).

### Scanning Electron Microscopy (SEM)

The supernatant was removed from each well and the biofilms on the titanium discs fixed in 2.5% (v/v) glutaraldehyde (Agar Scientific Ltd, Stansted, UK) solution in PBS for 1.5 h at 4°C. The samples were then washed (4 ×) using sterile dH_2_0 prior to being immersed in water (100 µl) and freeze-dried. The discs were sputter-coated with gold, prior to imaging using a TESCAN, VEGA3 SEM at 5.0 kV. Sterile titanium disc controls were also analyzed.

### Bacterial drop counts

Following treatment, the supernatant was removed from each well and the titanium discs washed with PBS. Each disc was transferred to a universal tube containing 5 ml PBS and agitated for 30 s on a vortex mixer and placed in a sonicating water bath for 5 min, followed by re-agitation on the vortex mixer for 2 min. Each bacterial suspension was serially diluted and plated onto BA or FAA plates prior to being incubated for 48 h (*S. mutans*) or 48–72 h (*P. gingivalis*), respectively. Counts (CFU/ml) for each dilution were then determined.

### Statistical analysis

MIC and MBEC values are presented as the mode of three biological repeats. Other data are presented as mean ± standard deviation (SD) from three biological repeats. Group-wise comparisons were analyzed by parametric one-way ANOVA followed by Dunnett’s multiple comparison tests. Statistical analysis was carried out using Graph Pad Prism® (GraphPad Software Inc., San Diego, CA, USA; v9.5.0), and statistical significance was assumed at *p* < 0.05.

## Results

### Epoxy-tigliane compounds demonstrate antimicrobial effects against Gram-positive and Gram-negative oral pathogens

The MIC and MBEC values for *S. mutans*, *A. actinomycetemcomitans* and *P. gingivalis* varied according to the epoxy-tigliane structure (Supplementary Table S1). For *S. mutans*, MICs of 32 µg/ml (EBC-1013), 256 µg/ml (EBC-46), and 1024 µg/ml (EBC-147) were obtained, whilst against *P. gingivalis* lower MICs of 8 µg/ml (EBC-1013) and 128 µg/ml (EBC-46) were achieved (with EBC-147 being the same). Due to the observed effect of the ethanol vehicle control (v/v) against *A. actinomycetemcomitans* (with inhibition at >256 µg/ml), valid MICs could not be reported for this strain. It was apparent that the EBC compounds demonstrated a hierarchy of antimicrobial efficacy against these oral strains, namely EBC-1013 > EBC-46 > EBC-147.

Against *S. mutans* and *P. gingivalis*, the MBEC values for EBC-1013 and EBC-46 were 2 and 4-fold greater, respectively, than the observed MIC values (Supplementary Table S1), although no effective difference was seen with EBC-147. MBECs against *A. actinomycetemcomitans* were observed at 1024 µg/ml and 512 µg/ml for EBC-1013 and EBC-147, respectively, with no MBEC obtained for EBC-46 (>1024 µg/ml). MBEC analysis on TiO_2_-coated PEG plates also showed that EBC-1013 (256 and 128 µg/ml) was more effective than EBC-46 (2048 and 512 µg/ml) against *S. mutans* and *P. gingivalis* biofilms, respectively. As previously, EBC-147 gave invalid MBEC results due to the inhibitory effect of the vehicle control (at >1024 µg/ml).

**Figure 2.**
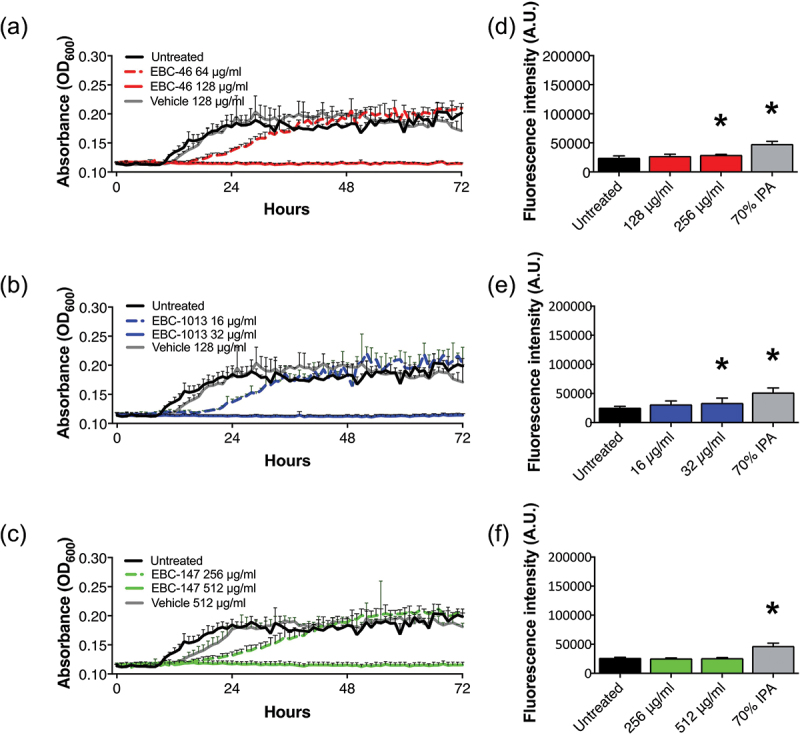
*P. gingivalis* (NCTC 11834) growth curves in FAB (72 h) ± (a) EBC-46, (b) EBC-1013 and (c) EBC-147 with untreated and vehicle equivalent controls (v/v). Effect of (d) EBC-46, (e) EBC-1013 and (f) EBC-147 on cell membrane permeabilization of *P. gingivalis* (NCTC 11834), with untreated and 70% isopropanol positive controls. Results are expressed as fluorescence intensity (A.U.; *p < 0.05).

The growth curves for *S. mutans* with EBC-46 and EBC-1013 showed virtually complete growth inhibition at the MIC value (Figure 1b–d; EBC-1013 > EBC-46 > EBC-147) and slight growth inhibition at ½MIC (EBC-1013 > EBC-46), with cell membrane permeabilization induced at one-fold > MIC value. No permeabilization was induced by EBC-147 (MIC >512 µg/ml) (Figure 1e–g). The growth curves for *P. gingivalis* demonstrated complete growth inhibition for EBC-46, EBC-1013 and EBC-147 at 128 µg/ml (MIC value), 32 µg/ml (two-fold > MIC value) and 512 µg/ml (one-fold < MIC value), respectively (Figure 2a–c). In contrast, effects at half these concentrations showed shallower exponential growth phase curves between 8 and 36 h, which subsequently (36–72 h) recovered to those of the controls. Similarly, the cells were permeabilized at one-fold > MIC value for EBC-46 and two-fold > MIC value for EBC-1013. EBC-147, however, failed to permeabilize the cells (Figure 2d–f). Results for *A. actinomycetemcomitans* growth curves were invalid because of the observed effect by the vehicle control (Figure S1A-C). The epoxy-tiglianes also failed to induce permeabilization in *A. actinomycetemcomitans* (Figure S1D-F).

### EBC-1013 induces an antibiofilm effect on biofilm formation and on established biofilms at clinically relevant concentrations

**Figure 3.**
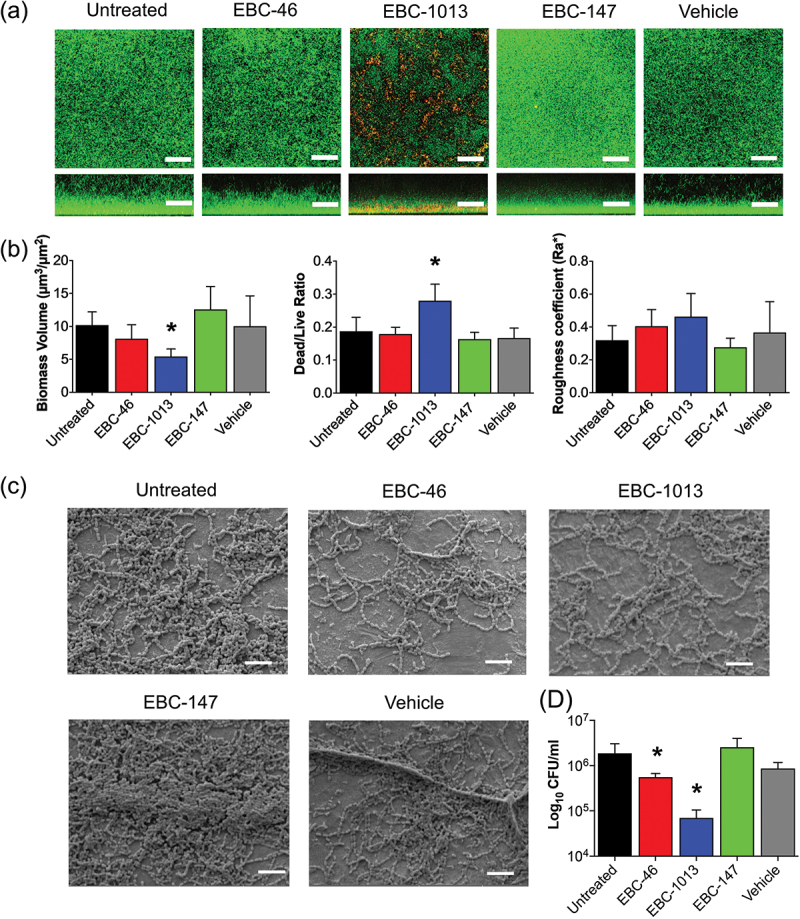
(a) Confocal laser scanning microscopy (CLSM) imaging (aerial and side views; scale bar 50 µm), with LIVE/DEAD® staining, of *S. mutans* (DSM 20523) 24 h biofilm formation in MH broth ± EBC treatment (256 µg/ml) with untreated and vehicle equivalent controls (v/v), (b) with corresponding COMSTAT image analysis of the biofilm CLSM z-stack images. (c) Scanning electron microscopy (SEM) imaging (scale bar; 20 µm) and (d) bacterial cell counts of established *S. mutans* (DSM 20523) biofilms (24 h) grown on titanium discs followed by a further 24 h ± EBC treatment (256 μg/ml) with untreated and vehicle controls (CFU/ml; *p < 0.05).

CLSM and COMSTAT image analyses revealed significantly reduced growth of *S. mutans* biofilms in biofilm formation assays compared to untreated and vehicle controls, typified by lower biofilm biomass volume and increased DEAD:LIVE cell ratio, observed for EBC-1013 tested at 256 µg/ml (*p* = 0.0001), with no significant effects observed for EBC-46 or EBC-147 (Figure 3a,b; *p* > 0.05). However, both EBC-1013 and EBC-46 treatment significantly disrupted established *S. mutans* (24 h) biofilms on titanium disc surfaces (Figure 3c). Plate count results (EBC-1013 = 6.9 × 10^4^ CFU/ml compared to 1.9 × 10^6^ CFU/ml for the untreated control; *p* = 0.002) were confirmed by SEM imaging of the titanium disc surfaces, with EBC-1013 and EBC-46 treatment again both reducing the biofilm biomass and the EBC-147 and vehicle controls failing to demonstrate any apparent effects (Figure 3d).

For *A. actinomycetemcomitans*, a significant decrease in biomass volume, as well as an increase in surface roughness and DEAD:LIVE cell ratio (*p* = 0.0001) was observed for EBC-1013 (256 µg/ml). EBC-46 had no effect on the *A. actinomycetemcomitans* biofilms, whilst EBC-147 exhibited a significant effect on biomass volume at 256 µg/ml (Figure S2A-B; *p* = 0.002).

**Figure 4.**
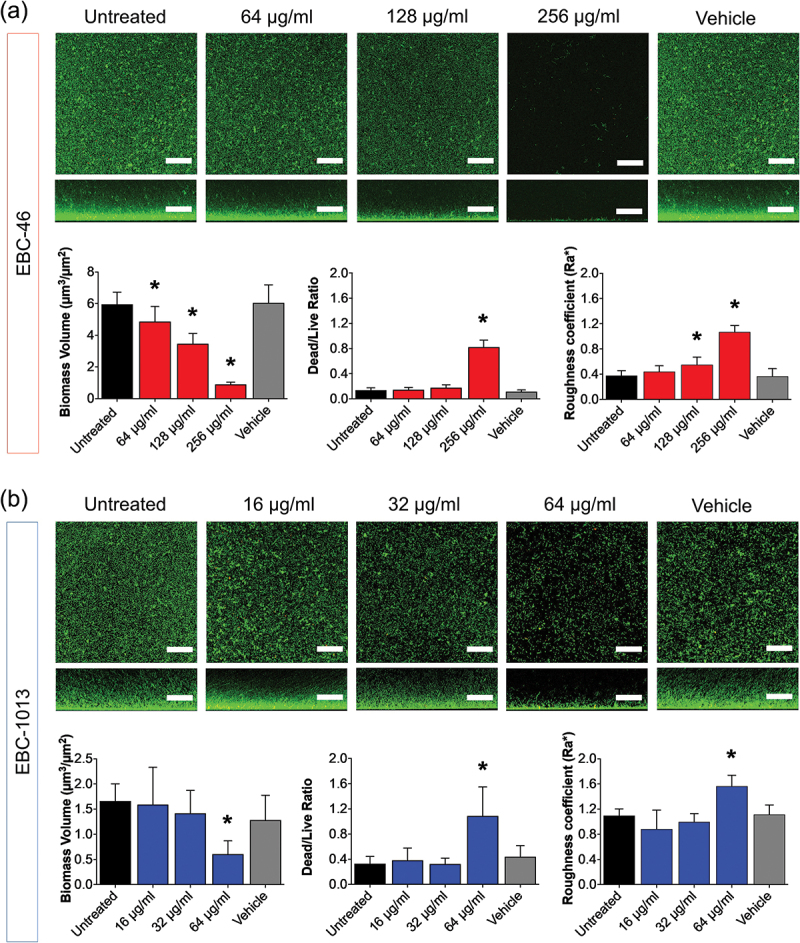
Confocal laser scanning microscopy (CLSM) imaging (aerial and side views; scale bar 50 µm), with LIVE/DEAD® staining, of *P. gingivalis* (NCTC 11834) 96 h biofilm formation in FAB ± (a) EBC-46 treatment (64–256 µg/ml) or (b) EBC-1013 (16–64 µg/ml) treatment with untreated and vehicle equivalent controls (v/v), and corresponding COMSTAT image analysis of the biofilm CLSM z-stack images (*p < 0.05; *n* = 3).

**Figure 5.**
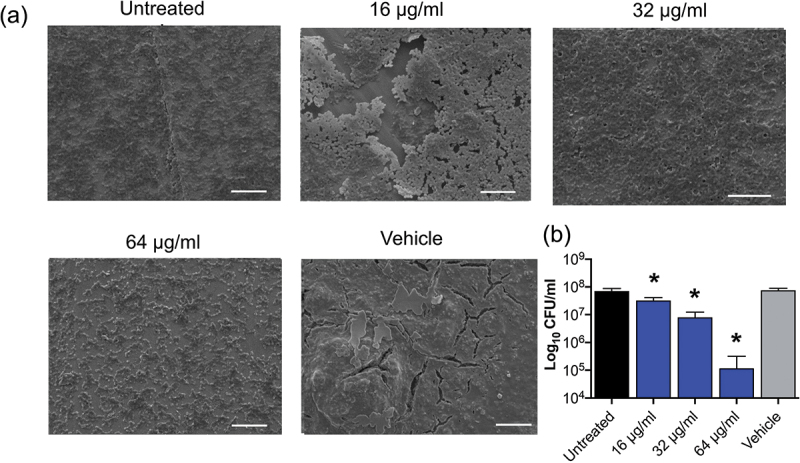
(a) Scanning electron microscopy imaging (scale bar; 10 µm) of established *P. gingivalis* (NCTC 11834) biofilms (96 h) grown on titanium discs followed by a further 24 h ± EBC-1013 (16–64 μg/ml) with untreated and vehicle controls (CFU/ml) with (b) corresponding bacterial cell counts (*p < 0.05).

Biofilm formation assays were conducted to determine the concentration-dependent effect of the two most potent antibiofilm EBC candidates on *P. gingivalis*; EBC-46 (64–256 µg/ml; MBEC 256 µg/ml) and EBC-1013 (16–64 µg/ml; MBEC 16 µg/ml). COMSTAT analysis of CLSM imaging demonstrated a concentration-dependent decrease in cellular aggregation. EBC-46 resulted in a statistically significant reduction in biomass volume at all concentrations tested, with a significant increase in DEAD:LIVE ratio at 256 µg/ml only (Figure 4a,b; *p* < 0.0001). EBC-1013 revealed a significant change in biomass volume and DEAD:LIVE ratio at 64 µg/ml, two-fold higher than the MBEC value (Figure 4c,d; *p* < 0.0001). A dose dependent effect of the lead candidate, EBC-1013, was further investigated using *P. gingivalis* (96 h) biofilms grown on titanium discs. SEM images and drop counts revealed the significant effect of EBC-1013 (≥16 μg/ml) against established biofilms on the titanium disc surface (Figure 5; *p* < 0.0001).

## Discussion

The antimicrobial activity of the semi-synthetic diterpene ester EBC-1013 and the naturally-occurring EBC-46 against a range of dental pathogens was demonstrated in the present study. The use of plant-derived compounds such as 0.03% sanguinarine and aloe as broad-spectrum antiseptics/disinfectants in dentistry is not new [23–25]. These products are typically non-specific in their mode of action, resulting in loss of structural organization, membrane damage and protein denaturation. The relative clinical efficacy of such antiseptics, however, is determined by the significant clinical properties of substantivity, penetrability and selectivity. Substantivity is the ability of an agent to bind to tissue surfaces, be released over a period of time to deliver an adequate dose of the active ingredient, and so result in activity necessary to confront the colonizing bacteria. Penetrability is the ability of the agent to infiltrate the formed bacterial matrix, and selectivity is the ability of the same agent to affect specific bacteria within. The challenge therefore remains to determine preliminary *in vitro* characteristics of the EBC structures prior to further clinical evaluation.

In this study, the ability of EBC-1013 to inhibit the growth of both Gram-positive and Gram-negative oral pathogens was evident. EBC-1013 consistently demonstrated increased *in vitro* antimicrobial activity when compared to EBC-46 (tigilanol tiglate), likely reflecting the contrasting molecular structure of EBC-1013, in line with previous studies using dermal wound pathogens (*Pseudomonas aeruginosa*, *Staphylococcus aureus* and *Escherichia coli*) [15]. EBC-46 and EBC-1013 were effective against *S. mutans* and *P. gingivalis* inducing both membrane permeabilization and reducing growth. Increased permeabilization by EBC-1013 (effectively permeabilizing *S. mutans* and *P. gingivalis* at ≤64 µg/ml) is likely indicative of the greater hydrophobicity of the EBC-1013 compound (with extended C-12 ester chains; log *P =* 2.37) versus EBC-46 (log *P* = 1.23) [15]. The failure of epoxy-tiglianes to induce statistically significant permeabilization in *A. actinomycetemcomitans* could result from the sensitivity of *A. actinomycetemcomitans* to the ethanol vehicle control (>256 µg/ml). In contrast to the Gram-negative chronic wound isolates tested in Powell et al. (2022) [15], MICs were obtained for *P. gingivalis*. This finding could be due to slow bacterial growth under anaerobic conditions; oxygen availability having previously been shown to affect the antimicrobial efficacy of antimicrobials such as peptide piscidin–copper interactions [26]. The mechanism of action of the epoxy-tiglianes is unclear and may represent PKC modulation known to be important in mediating the anticancer activity of tigilanol tiglate [27]. Here, EBC-1013 and EBC-46 (with similar PKC-stimulating activity) were effective against both Gram-positive *S. mutans* and Gram-negative *P. gingivalis*. In contrast, EBC-147 with low PKC-inducing activity, exhibited minimal anti-microbial activity. Whilst the precise role of PKC activation in bacteria is unclear, precursors of PKC isotypes in *E. coli* have shown phospholipid- and Ca^2+^-dependent phorbol ester binding [28]. The potential role of PKC in this process is supported by the very low antimicrobial activity of EBC-147, as previously observed against wound pathogens [15], mirroring its reduced ability to induce PKC activation [27].

In addition to the antimicrobial effects in planktonic systems, the antibiofilm activity of EBC-1013, EBC-46 and EBC-147 against oral biofilms was also studied. Biofilms are implicated in up to 80% of all microbial infections [29]. Reduced metabolic activity within the biofilm, and the host- and bacterially derived extracellular ‘polymer mesh’ confers increased resistance to chemical and mechanical disruptions of the structure [30]. The superior antibiofilm activity of EBC-1013 (compared to EBC-46 and EBC-147) was evident for both Gram-positive *S. mutans* and Gram-negative *P. gingivalis* in this study, demonstrating reduced biofilm bio-volume as well as a reduction in colonization of titanium surfaces following EBC-1013 treatment. It has been hypothesized that the direct antibiofilm activity of EBC-1013 reflects biofilm extracellular polysaccharide (EPS) matrix disruption. Interestingly, previous studies have demonstrated how therapeutic disruption of biofilm structures may effectively reduce the elasticity (Young’s modulus) of biofilms and increase susceptibility to hydrodynamic shear [31], both of which may be important in attempts to effectively eliminate the biofilm at the host/implant interface in peri-implantitis.

In peri-implantitis, extracellular matrix (ECM) remodeling will occur following surgical debridement [32]. EBC-1013, being a PKC activator (and potentially other C1 domain-containing proteins), exhibits immunomodulatory activity [15]. Interestingly, in diabetic skin wounds, EBC-1013 was able to induce a local inflammatory response, with *Tnf, Il1b, Il6, Il36g*, and *Cxcl2* induction and polymorphonuclear leukocyte (PMNL) recruitment, as well as reorganization and remodeling of the extracellular matrix, and wound re-epithelialization. The immunostimulatory activity of EBC-1013 contrasts with the immunomodulatory activities of peptides such as pleurocidin [33], and essential oils (already in use in dentistry) [34], which inhibit inflammatory responses by suppressing the production of important mediators of pro-inflammatory pathways [35–37]. Innate immune induction by EBC-1013 induced healing in 6/7 diabetic wounds, compared to only 1/7 of untreated wounds. The induction of a rapidly resolving local innate immune response, combined with rapid remodeling at the implant/host interface, may contribute to the removal of biofilm persister cells from the sites of debridement.

The safety profile of EBC-1013 is important to consider in the translation to clinical use. EBC-1013 exhibited antimicrobial activity against oral pathogens in MICs and MBECs in this study at dose levels suitable for injection into diseased peri-implant or periodontal tissues. Phase I human safety/dose-escalation studies failed to demonstrate a maximum tolerated dose of intradermal injection using EBC-46 [38] at 1 mg/ml, with only mild and transient side-effects reported [39].

Mechanical disruption and removal of the biofilm represents the ‘gold standard’ strategy for the treatment of the peri-implantitis [40], although confounding factors such as implant geometry and surface topography may render mechanical cleaning alone insufficient [41]. Whilst these data with EBC-1013 are encouraging as a topical treatment alongside mechanical debridement for peri-implantitis, future work will involve testing antimicrobial activity against a wider range of pathogens associated with peri-implantitis, e.g. *Tannerella forsythia* and *Prevotella intermedia* [42], and will investigate the antimicrobial effect of EBC-1013 in multispecies oral biofilm models. Furthermore, confirming the structure activity relationships of the different epoxy-tigliane structures could broaden the opportunities available for tailoring products towards other chronic oral inflammatory diseases such as periodontitis.

## Conclusion

These *in vitro* data provide evidence of the antimicrobial activity of EBC-1013 against Gram-positive and Gram-negative oral pathogens at doses readily identified for *in vivo* use at the host-implant interface. The antibacterial activity is mirrored by the antibiofilm effects. These effects, coupled with the known ability of the agent to induce self-resolving innate immune system activation, represent a completely novel approach to disrupt the biofilm in peri-implantitis and enhance the effects of mechanical debridement.

## Supplementary Material

Supplemental Material
