# Supplementary material for: Defining in vitro topical antimicrobial and antibiofilm activity of epoxy-tigliane structures against oral pathogens
Source: J Oral Microbiol. 2023 Jul 31;15(1):2241326. doi: 10.1080/20002297.2023.2241326 (PMC10392292; doi:10.1080/20002297.2023.2241326)
Supplement: Supplemental Material [file ZJOM_A_2241326_SM8584.zip › Supplementary files/SupplementaryTable1.docx]

**TABLE S1** Minimum Inhibitory Concentration (MIC) and Minimum Biofilm Eradication Concentration (MBEC) values (µg/ml) for EBC-46, EBC-1013 and EBC-147 (including vehicle controls) against oral pathogens *S. mutans* DSM 20523, *A. actinomycetemcomitans* DSM 8324 and *P. gingivalis* NCTC 11834.

|  |  | | **MIC and MBEC at μg/ml (or vehicle v/v equivalent)** | | | |
| --- | --- | --- | --- | --- | --- | --- |
| **Bacterial strain** | **Growth conditions** | **Test** | **EBC-1013** | **EBC-46** | **EBC-147** | **Vehicle control** |
| *S. mutans*  DSM 20523 | 5% CO_2_ | MIC  MBEC | 32  64 | 256  1024 | 1024  >1024^a^ | >1024  >1024^a^ |
| *A. actinomycetemcomitans*  DSM 8324 | 5% CO_2_ | MIC  MBEC | 512^a^  1024 | 512^a^  >1024^a^ | 512^a^  512 | >256  >1024 |
| *P. gingivalis*  NCTC 11834 | Anaerobic | MIC  MBEC | 8  16 | 128  256 | 1024  1024 | >1024  >1024 |
| *S. mutans*  DSM 20523 | TiO_2_-coated PEG | MBEC | 256 | 2048 | >1024^a^ | >1024 |
| *P. gingivalis*  NCTC 11834 | TiO_2_-coated PEG | MBEC | 128 | 512 | 1024 | >1024 |

^a^Indicates invalid MIC/MBEC result as the vehicle control was also positive at this concentration.

^b^MBECs were performed using traditional microtitre plate assays and on titanium oxide (TiO_2_)-coated PEG (Calgary device) plates.
