# Supplementary figures and images for: Defining in vitro topical antimicrobial and antibiofilm activity of epoxy-tigliane structures against oral pathogens
Source: J Oral Microbiol. 2023 Jul 31;15(1):2241326. doi: 10.1080/20002297.2023.2241326 (PMC10392292; doi:10.1080/20002297.2023.2241326)

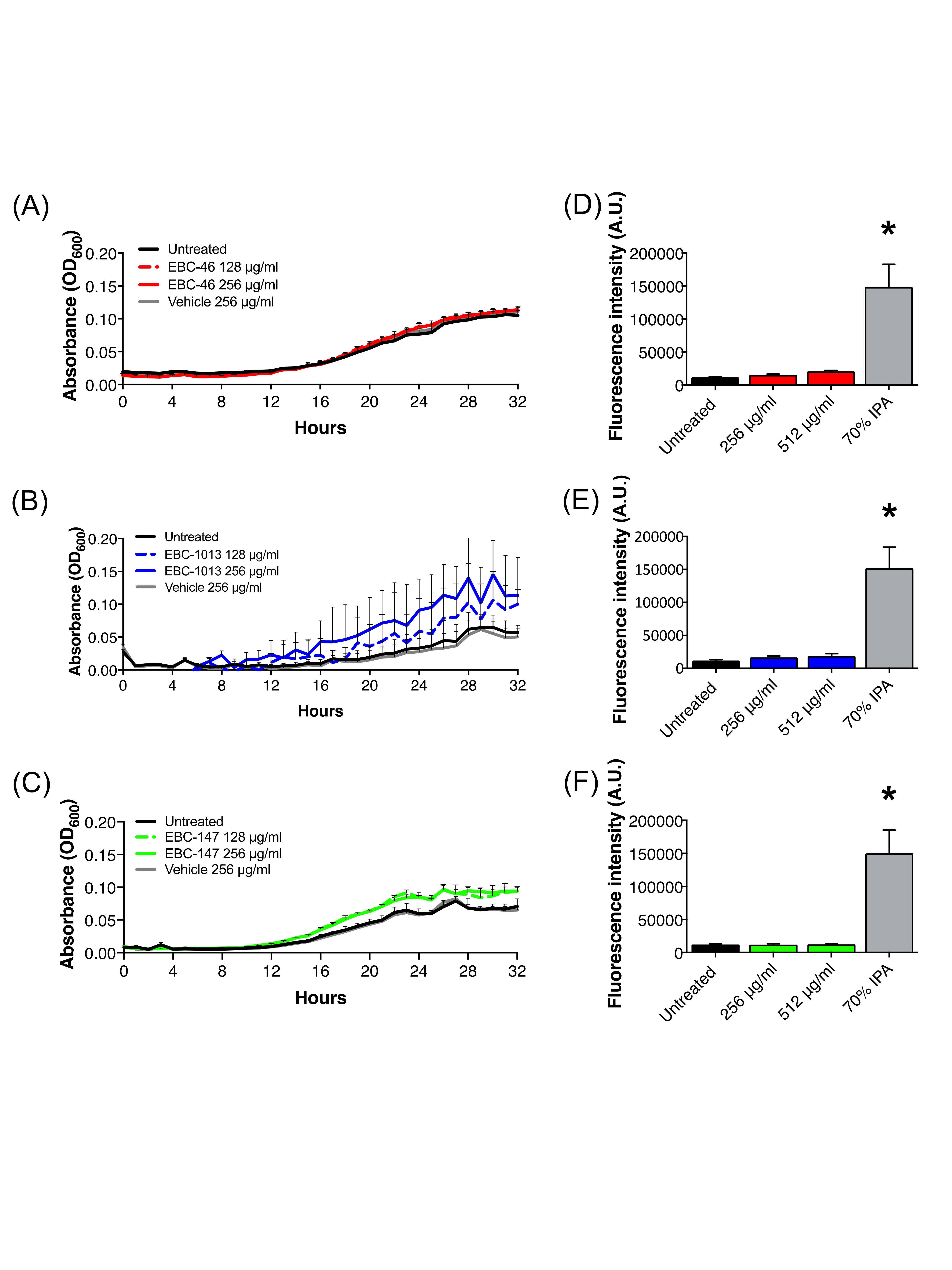

Supplement: Supplemental Material [file ZJOM_A_2241326_SM8584.zip › Supplementary files/SupplementaryFigure_1.jpg]

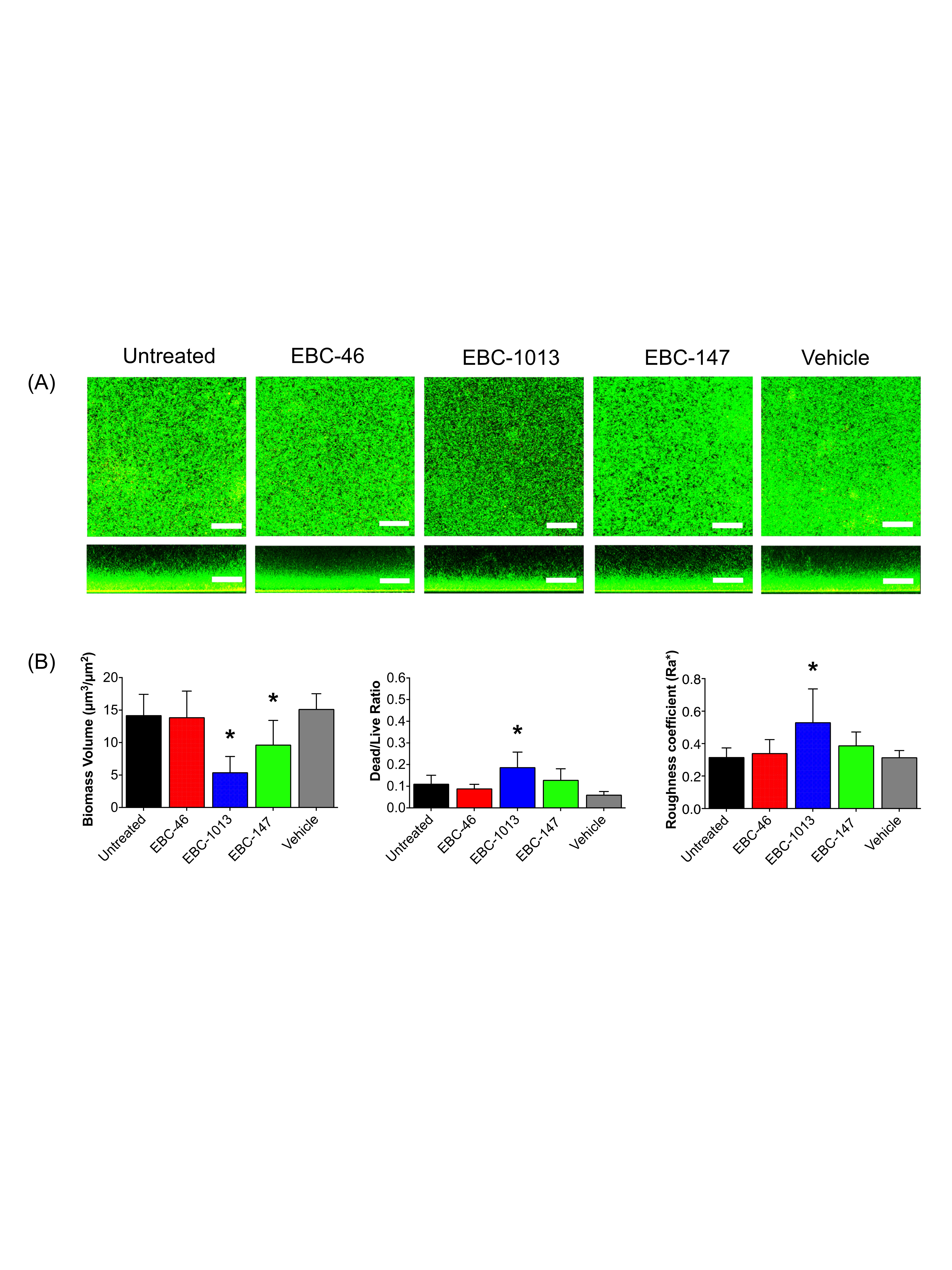

Supplement: Supplemental Material [file ZJOM_A_2241326_SM8584.zip › Supplementary files/SupplementaryFigure_2.jpg]
